# A Case of RhabdomyoSarcoma Following a Metal Surgical Implant

**DOI:** 10.1080/13577149977794

**Published:** 1999-09

**Authors:** Giovanna M. Gatti, Giovanni B. Ivaldi, Eric Lartigau, Hugo Marsiglia, Roberto Orecchia

**Affiliations:** 1 Division Radiotherapy European Institute of Oncology via Ripamonti 435 Milan I-20141 Italy; 2 Division Radiotherapy U.Z. Gasthuisberg Herestraat 49 Leuven Belgium; 3 Chair of Radiotherapy University of Milan Italy; 4 Institute Gustave Roussy Villejuif France

## Abstract

*Patient.* We report a 51-year-old male presenting with Grade III rhabdomyosarcoma.

*Discussion.* A case of rhabdomyosarcoma which developed in proximity to a metal surgical implant is described. Few cases have been reported in the world in humans.The therapeutic approach to the disease is presented, together with a brief review of literature.


*Sarcoma* (1999) 3, 145–147

CASE REPORT

### A case of rhabdomyosarcoma following a metal surgical implant

GIOVANNA M. GATTI,[1,2] GIOVANNI B. IVALDI,[1] ERIC LARTIGAU,[4]
HUGO MARSIGLIA[1] & ROBERTO ORECCHIA[1,3]

[1]*Division Radiotherapy, European Institute of Oncology, via Ripamonti 435, Milan, Italy,* [2]*Division Radiotherapy, U.Z. Gasthuisberg, Herestraat 49, Leuven, Belgium,* [3]*Chair of Radiotherapy, University of Milan, Italy, &* [4]*Institute Gustave Roussy, Villejuif, France*

**Abstract**
*Patient.* We report a 51-year-old male presenting with Grade III rhabdomyosarcoma.
*Discussion.* A case of rhabdomyosarcoma which developed in proximity to a metal surgical implant is described. Few cases have been reported in the world in humans. The therapeutic approach to the disease is presented, together with a brief review of literature.

**Key words:** *rhabdomyosarcoma, metal surgical implant.*

### Introduction

The etiology of soft tissue sarcomas is not well defined. Biomaterials, such as metals and metallic alloys, synthetic and natural materials, can be considered potential physical carcinogens possibly involved in some cases of soft tissue sarcoma.[1–5] Malignant tumors around fracture fixation implants have been reported for many years. The first description of sarcoma developing in proximity to a biomaterial was made by Turner, who found that Bakelite disks, implanted in rats, were able to cause fibrosarcomas.[2] Earlier, anecdotal cases of tumors that arose around foreign bodies (i.e. bullets) had been reported. Other more recent case reports have discussed the possible relation between the surgical implantation of metals and the development of different forms of sarcoma.[6–10]

The studies performed to demonstrate the carcinogenicity of hard and soft materials are nearly exclusively experimental: metals have been evaluated as potential carcinogens by administering pure elements or compounds by a variety of routes, such as oral administration, inhalation, implantation in the peritoneum, pleura or parenteral injection. Rodents have been used in many of these studies.[11–14] Fibrosarcoma and rhabdomyosarcoma are the most common sarcomas to develop in these experiments.[15] Maltoni has suggested that proliferation of mesenchymal cells found in the fibrous foreign body reaction to implanted biomaterials is the initiating event in the development of the sarcoma.[16]

### Case report

Mr S.A., 51 years of age, suffered a fracture of the right femur in 1985 and underwent a surgical implant of a metal prosthesis. In 1993 he began to complain of an acute pain in the right thigh, over the months the symptoms progressed to include the entire right leg and in 1995 it also involved the perineum. In February 1995 he had the synthetic inserts in the right femur surgically removed. An ultrasound of abdomen and pelvis proved negative.

In January 1996 the patient was hospitalised. An ultrasound of the right gluteus showed the presence of a large fluid mass of irregular shape and density, with a diameter of 9×5×15 cm. A biopsy was performed and a histologic diagnosis of ‘Grade III Rhabdomyosarcoma’ was made. An X-ray of the chest also demonstrated the presence of bilateral lung metastases.

On CT the neoplastic mass appeared to be situated in front of the sacrum: it was displacing the rectum, the urethra and the urinary bladder and was close to the site of the metal implant (Fig. 1). The patient received treatment with chemotherapy, palliative surgery and radiation. Unfortunately, cerebral metastases developed and the patient died 10 months after diagnosis.

### Discussion

In the clinical case presented, the rhabdomyosarcoma developed in proximity to a stainless steel

Correspondence to: Giovanna M. Gatti, Radiotherapy Division, European Institute of Oncology, via Ripamonti 435, I-20141 Milan, Italy. E-mail: ggatti@zdnetmail.com

1357-714X print/1369-1643 online/99/020145-03 © 1999 Taylor & Francis Ltd

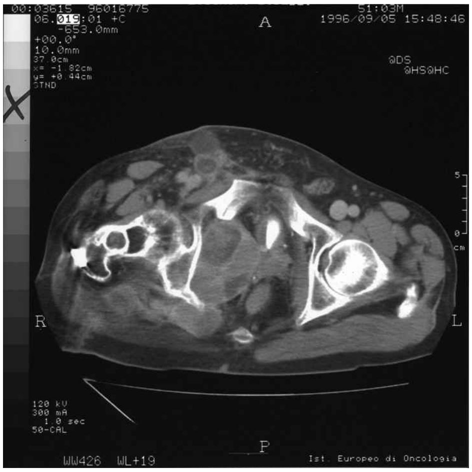

**Fig. 1.** *A CT-scan image showing the proximity of the tumor mass to the area of hip replacement.*

implant (Fig. 1). Although the relationship between surgical implants and sarcogenesis has not been clearly demonstrated, several case reports have described a temporal relationship between the placement of surgical implants and the successive development of sarcoma.[17,18] The most frequent histotype described was fibrosarcoma[19,20] with rhabdomyosarcoma also described.[21]

Recently a patient was reported to have a malignant fibrous histiocytoma in the bone–cement membrane after revision for a loose total hip prosthesis. Existing biologic reports on the response of mesenchymal cells to metallic debris seem to suggest that the environment surrounding a loosened prosthesis may provide conditions appropriate for the development of a sarcoma. Because of the similarity on plain radiograms of sarcomatous lesions to lesions known to be caused by wear debris, tumors should be included in the differential diagnosis of cases of total hip loosening.[22,23]

A report by Penman *et al.*[24] describes the occurrence of an osteosarcoma at the site of a cobalt–chrome total hip replacement; the possibility of the tumor arising as a result of the liberation of cobalt particles is discussed in the same report. A case report on a synovial sarcoma developed on a total hip replacement has been published in 1988;[25] a case of angiosarcoma associated with a Dacron graft has also been reported.[26] A study of 1996 used two groups of patients to verify the incidence of cancer after metal on metal total hip arthroplasty and metal on polyethylene total hip arthroplasty compared with the incidence of cancer in the general population in Finland. The result was that the risk of total cancer in the patients who had metal on metal total hip arthroplasty was 1.23-fold compared with that of the patients who had polyethylene on metal total hip arthroplasty. No sarcomas were observed at the site of the prosthesis.[27]

Sarcoma developing in proximity to a metallic bioimplant is a rare occurrence. In the future however, an ageing population with increased longevity and a higher prevalence of arthroplasty surgery may increase the frequency that this condition is encountered.

### References

1 Shiffman MA. Breast implants and cancer (letter; comment). Comment on: *J Natl Cancer Inst* 1997; 89(18):1341–9.
2 Iomhair MM, Lavelle SM. Effect of film size on production of foreign body sarcoma by perforated film implants. *Technology & Health Care* 1997; 5(4):331–4.
3 Turner FC. Sarcomas at sites of subcutaneous implanted Bakelite disks in rats. *JNCI* 1941; 2:81.
4 Morgan RW, Elcock M. Artificial implants and soft tissue sarcomas. *Journal of Clinical Epidemiology* 1995; 48(4):545–9.
5 Weber PC. Epithelioid sarcoma in association with total knee replacement. A case report. *Journal of Bone & Joint Surgery—British Volume* 1986; 68(5):824–6.
6 Ward JJ, Thornbury DD, Lemons JE, Dunham WK. Metal-induced sarcoma. A case report and literature review. *Clin Orthop* 1990; 299–306.
7 Tayton KJ. Ewing's sarcoma at the site of a metal plate. *Cancer* 1980; 45(2):413–5.
8 Lindeman G, McKay MJ, Taubman KL, Bilous AM. Malignant fibrous histiocytoma developing in bone 44 years after shrapnel trauma. *Cancer* 1990; 66(10):2229–32.
9 Bell RS, Hopyan S, Davis AM, Kandel R, Gross AE. Sarcoma of bone–cement membrane: a case report and review of the literature. *Can J Surg* 1997; 40(1):51–5.
10 Jacobs JJ, Rosenbaum DH, Hay RM, Gitelis S, Black J. Early sarcomatous degeneration near a cementless hip replacement. A case report and review. *Journal of Bone & Joint Surgery—British Volume* 1992; 74(5):740–4.
11 Maltoni C, Sinibaldi C. Carcinogenicity of acrylic resins (polymethyl methacrylate) used in dentistry. Long-term bioassays on Sprague–Dawley rats by subcutaneous implantation. *Acta Oncol* 1982; 3:13.
12 Maltoni C, Sinibaldi C, Morisi L. Carcinogenicity of vitallium: long-term bioassays on Sprague–Dawley rats and Swiss mice by subcutaneous implantation. *Acta Oncol* 1980; 1:11.
13 Oppenheimer BS, Oppenheimer ET, Danishefsky I, Stout AP. Carcinogenic effect metals in rodents. *Cancer Res* 1956; 16:439.
14 Oppenheimer BS, Oppenheimer ET, Stout AP, Danishefsky I. Malignant tumours resulting from embedding plastics in rodents. *Science* 1953; 118:305.
15 Furst A. Bioassay of metals for carcinogenesis: whole animals. *Environ Health Perspect* 1981; 40:83–91.
16 Maltoni C, Santi L, Del Gaudio A. Sarcogenesi del tessuto sottocutaneo nel ratto da impianto di dischi di Teflon: sequenza delle modificazioni locali. (Sarcogenesis in subcutaneous tissue of the rat from implant of Teflon disque's: local modifications sequence.) *Il Cancro* 1964; 17:4.
17 Brien WW, Salvati EA, Healy JH, Bansal M, Ghelman B, Betts F. Osteogenic sarcoma arising in the area of a total hip replacement. A case report. *Journal of Bone & Joint Surgery—American Volume* 1990; 72(7):1097–9.
18 Hamblen DL, Carter RL. Sarcoma and joint replacement. *Journal of Bone & Joint Surgery—British Volume* 1984; 66(5):625–7.
19 Burns WA, Kanhouwa S, Tillman L, Saini N, Herrmann JB. Fibrosarcoma occurring at the site of a plastic vascular graft. *Cancer* 1972; 29(1):66–72.
20 Eckstein FS, Vogel U, Mohr W. Fibrosarcoma in association with a total knee joint prosthesis. *Virchows*

*Archiv—A, Pathological Anatomy & Histopathology* 1992; 421(2):175–8.

21 Kreicbergs A. Pseudotumor after metal fixation of a fracture surgery. A case report. *Acta Orthop Scand* 1983; 54(5):739–42.

22 Ryu RK, Bovill EG Jr, Skinner HB, Murray WR. Soft tissue sarcoma associated with aluminium oxide ceramic total hip arthroplasty. A case report. *Clinical Orthopaedics & Related Research* 1987; (216):207–12.

23 Van Odijk J, Marchal G, Baert A, Mulier J, Verhelst M. The radiological images of complications of the total hip arthroplasty. *Journal Belge de Radiologie* 1974; 57(6):429–36.

24 Penman HG, Ring PA. Osteosarcoma in association with total hip replacement. *Journal of Bone & Joint Surgery—British Volume* 1984; 66(5):632–4.

25 Lamovec J, Zidar A, Cucek-Plenicar M. Synovial sarcoma associated with total hip replacement. A case report. *Journal of Bone & Joint Surgery—American Volume* 1988; 70(10):1558–60.

26 Fehrenbacher JW, Bowers W, Strate R, Pittman J. Angiosarcoma of the aorta associated with a Dacron graft. *Annals of Thoracic Surgery* 1981; 32(3):297–301.

27 Visuri T, Pukkala E, Paavolainen P, Pukkinen P, Riska EB. Cancer risk after metal on metal and polyethylene on metal total hip arthroplasty. *Clin Orthop* 1996; (329 Suppl):S280–9.
